# Host-microbiota interactions in the infant gut revealed by daily faecal sample time series

**DOI:** 10.20517/mrr.2024.45

**Published:** 2024-12-27

**Authors:** Nienke van Beek, Iiris Katavisto, Markku Lehto, Kaija-Leena Kolho, Willem M. de Vos, Anne Salonen, Katri Korpela

**Affiliations:** ^1^Human Microbiome Research Program, Faculty of Medicine, University of Helsinki, Helsink 00014, Finland.; ^2^Folkhälsan Institute of Genetics, Folkhälsan Research Center, Helsinki 00250, Finland.; ^3^Research Program for Clinical and Molecular Metabolism, University of Helsinki, Helsinki 00014, Finland.; ^4^Department of Nephrology, University of Helsinki and Helsinki University Hospital, Helsinki 00014, Finland.; ^5^Faculty of Medicine, University of Helsinki and Children’s Hospital, Helsinki University Hospital HUS, Helsinki 00014, Finland.; ^6^Laboratory of Microbiology, Wageningen University, Wageningen 6700 EH, the Netherlands.; ^7^Department of Bacteriology and Immunology, Faculty of Medicine, University of Helsinki, Helsink 00014, Finland.

**Keywords:** Infant gut microbiome, immune biomarkers, IAP, mucin, lipocalin 2, albumin

## Abstract

**Aim:** This study aims to explore the interplay between host immune factors and gut microbiota in human infants *in vivo* using time-series daily stool samples and identify biomarkers of host-microbe interactions.

**Methods:** 216 faecal samples collected from infants aged 5-6 or 11-12 months were analysed for gut microbiota composition, total bacterial load, and biomarkers of immune function.

**Results:** We identified indications of microbial stimulation of eosinophil cationic protein (ECP), IgA, calprotectin (Cal), intestinal alkaline phosphatase (IAP), and Bactericidal/permeability-increasing protein (BPI) at 6 and 12 months, as well as stimulation of lipocalin 2 (LCN2), lactoferrin (LTF), and alpha-defensin-5 only at 6 months. The associations between biomarker concentrations and bacterial population growth were primarily positive at 6 months and mostly negative at 12 months, suggesting increasing host regulation of the microbiota with age. The exceptions were IAP, which was predictive of declining bacterial populations at both time points, and Cal, whose associations changed from negative at 6 months to positive at 12 months.

**Conclusion:** There is an age-associated development in the correlation pattern between bacterial population growth and the biomarker concentrations, suggesting that host-microbe interactions change during early development. Albumin appeared as a potential marker of gut permeability, while LCN2 seemed to correlate with gut transit time. Mucin degradation appeared to decrease with age. Mucin2 and IAP emerged as potentially important regulators of the bacterial populations in the infant gut. The study demonstrates the utility of biomarker and bacteria profiling from daily stool samples for analysing *in vivo* associations between the immune system and the gut microbiota and provides evidence of host regulation of the microbiota in infants.

## INTRODUCTION

The human body is a bustling metropolis of microorganisms collectively known as the microbiota. The microbiota includes fungi, bacteria, archaea, and bacteriophages, with bacteria forming the most abundant taxonomic group. In the human gut, the main bacterial species are members of the phyla *Firmicutes*, *Bacteroidetes*, *Proteobacteria*, *Actinobacteria*, and *Verrucomicrobia*^[1]^.

The microbiota of an individual begins to form at birth, with significant colonisation happening at the moment of birth. During vaginal birth, the infant receives microbes from the mother, which lays the groundwork for microbiota development^[2]^. After the initial seeding, the composition of the gut microbiome is influenced by everything the infant is exposed to, mainly what they consume, including solid foods^[3,4]^.

The gut microbiome is involved in many vital facets of life, such as digestion and regulation of metabolism and the immune system^[5]^. Dysregulation of the microbiota is associated with a wide range of diseases, both during infancy and later in life, including metabolic diseases, allergic and chronic inflammatory diseases^[5,6]^.

Infants are born with an immature immune system^[7]^, making them vulnerable to infectious diseases within the first months of life. The infant gut microbiota can prevent pathogen colonisation and help train the immune system. Importantly, the infant gut microbiota impacts the later health of the host by affecting the development of the host’s physiology. Aberrations in the infant gut microbiota development are linked to the later onset of immune disorders such as atopic dermatitis (eczema), inflammatory bowel disease (IBD), and asthma, as well as obesity^[5,8]^. Because factors influencing the infant’s gut microbiome also impact the infant’s future health, ensuring a healthy infant microbiome, regardless of external factors such as birth method and feeding style, can be a cost-effective and promising approach to preventing later health issues.

To regulate the infant microbiome for health purposes, understanding the determinants of microbiota composition is essential. Most of the interindividual variation in gut microbiota composition in infants is still unexplained - studies have shown that approximately 20%-30% of the variation can be explained by external factors, such as birth mode, diet, and antibiotic exposure^[9,10]^. It is assumed that host-specific factors are important in regulating the gut microbiota, but these are poorly understood. The host influences the microbiome through dietary exposures and genetic factors. Quantitative trait loci (QTLs) that affect the microbiome are immune- or metabolism-related. Notably, the ABO (A, B antigens) and LCT locus (lactase) explain a small percentage of microbiota variance consistently across multiple GWAS^[11]^. How the gut microbiota interacts with the infant’s immature immune system and how the immune system, in turn, affects the abundance and composition of the microbiota have not been extensively studied.

Most human microbiota studies rely on cross-sectional data on the relative abundances of microbes. However, microbial populations undergo fluctuations in size, which may induce noise into cross-sectional data sets. Furthermore, compositional data suffer from the problem that the relative abundances of the different microbes are not independent, and thus, a change in one microbe will cause artefactual changes in other microbes^[12,13]^. True population growth or decline cannot be measured from relative abundance data. Analysing absolute abundances can overcome the problem of compositionality^[12,13]^.

Studies aimed at elucidating the link between immune markers and microbiota are often limited to a few markers or microbes and are performed in mice or *in vitro* or linked to a specific disease. As a result, we lack data on host-microbe interactions in healthy infants. This exploratory study aims to provide insight into the host factors influencing the microbiota and vice versa. We analysed stool samples from 6- and 12-month-old infants for 30 days. Stool biomarkers are reliable and non-invasive indicators of intestinal and, in some cases, general health^[14]^. We combined absolute abundances of bacteria based on metagenomic sequencing and qPCR with immune-related biomarkers: intestinal alkaline phosphatase (IAP) and bactericidal/permeability-increasing protein (BPI) as markers of host reaction to bacterial lipopolysaccharide that could inhibit bacterial growth^[15,16]^, human alpha defensin 5 (HD-5) as a marker of Paneth cell response^[17]^, eosinophil cationic protein (ECP) as a marker of eosinophil response^[18]^, lipocalin 2 (LCN2), lactoferrin (LTF), and calprotectin (Cal) as markers of inflammation and neutrophil response^[19,20]^, immunoglobulin A (IgA) as a general regulator of microbiota homeostasis in the gut^[15,21]^, mucin 2 (Muc2) as an indicator of mucus production^[22]^, and albumin as a potential indicator of gut epithelial integrity^[23]^. In addition, we measured faecal pH, total bacterial load using qPCR, and assessed the Bristol score. The daily samples enabled correlative analysis of the daily changes of both biomarkers and microbiota, enabling the identification of potential microbe-induced expression of the biomarkers and biomarker-induced regulation of the microbiota.

## METHODS

### Samples collection

Faecal samples of infants were collected as part of the Helmi Plus study in 2017-2018 as part of the HELMi cohort^[24]^. HELMi cohort consists of 1,055 healthy term infants born in 2016-2018, mainly in the capital region of Finland, and their parents. The intestinal microbiota development of the infants is characterised based on nine strategically selected faecal samples and connected to extensive online questionnaire-collected metadata at weekly to monthly intervals focusing on the diet, other exposures, and family’s lifestyle, as well as the health and growth of the child. A subset of the HELMi families participated in HelmiPlus, where the caretakers collected daily samples for 20-30 days when the infants were 5-6 months old (during the first introduction of solid foods) and/or 11-12 months old (when the infants were mostly consuming solid foods). Samples were stored in the home freezer at -20 °C until transported frozen to the lab and stored at -80 °C. This study used 216 faecal samples from 6 infants: 102 11-12-month-old (“12-month” time series) samples and 114 5-6-month-old (“6-month” time series) samples [Table 1]. Two babies have both a 6-month and a 12-month series in this sample set. All infants were breastfed at 5-6 months, and three infants still at 11-12 months. Three of the infants were born vaginally and three by Caesarean delivery. The infants were selected to represent a broad range of microbiota compositions at both 6 and 12 months and to have both birth modes represented.

**Table 1:** Median (interquartile range) biomarker concentrations categorised by birth mode and age

	**5-6 months**		**11-12 months**		**Total**	
	**Vaginal**	**C-section**	**Vaginal**	**C-section**	**Vaginal**	**C-section**
**Number of samples**	54	60	48	54	102	114
**IAP mg/g**	25.97 (6.20-58.85)	24.18 (16.32-32.73)	14.09 (8.28-23.19)	8.85 (3.14-16.07)	14.4 (3.9-32.7)	20.6 (10.1-29.7)
**BPI ng/g**	0.00 (0.00-0.00)	0.00 (0.00-0.00)	25.48595 (1.67-42.25)	0.00 (0.00-10.45)	0.00 (0.00-27.3)	0.00 (0.00-7.0)
**HD5 pg/g**	0.00 (0.00-142.21)	37.50 (0.00-230.32)	0.00 (0.00-34.46)	102.21 (52.49-160.04)	0.00 (0.00-73.2)	96.0 (0.00-184)
**ECP pg/g**	183.97 (0.00-1,037.84)	198.38 (0.00-558.94)	155.03 (0.00-331.03)	662.31 (488.187-772.91)	183 (0.00-684)	458 (173-672)
**IgA μg/g**	238.47 (54.26-609.15)	328.80 (227.96-452.58)	253.88 (123.26-1,031.91)	307.57 (208.23-497.60)	243 (80.6-905)	309 (208-491)
**LTF μg/g**	6.91 (2.53-12.25)	10.42 (6.49-16.08)	0.91 (0.34-2.69)	3.96 (1.90-6.68)	2.75 (0.89-9.11)	6.75 (3.75-12.40)
**Alb ng/g**	2.32 (0.33-20.63)	5.575 (2.74-25.19)	58.07 (7.31-198.96)	9.51 (6.39-17.34)	12.3 (1.91-72.1)	8.89 (4.50-22.6)
**LCN2 ng/g**	1.16 (0-5.47)	1.77 (0.00-17.51)	0.01 (0.00-22.70)	0.00 (0.00-1.24)	0.83 (0.00-8.95)	0.00 (0.00-6.55)
**Cal ng/g**	354.20 (88.01-1,602.02)	1,857.89 (1,199.08-2,707.68)	167.22 (0.00-465.38)	669.41 (266.36-1,958.44)	231 (21.0-842)	1,341 (601-2,542)
**Tot.prot. mg/g**	3.76 (0.31-13.79)	6.95 (0.00-17.16)	5.34 (0.87-8.37)	15.35 (11.40-18.67)	5.17 (0.46-11.8)	13.1 (6.16-17.6)
**Muc2 ng/g**	0.71 (0.30-2.42)	45.04 (22.53-75.84)	1.96 (0.20-7.41)	3.87 (1.75-7.99)	0.98 (0.24-5.81)	14.9 (4.00-51.5)
**Lysozyme ng/g**	0 (0-2.05)	0.00 (0.00-0.00)	0.00 (0.00-0.00)	0.9780727 (0-2.07)	0 (0-1.13)	0 (0-1.74)
**Zonulin ng/g**	0.0 (0.0-0.34)	0.7 (0.0-1.30)	1.5 (0.8-1.80)	0 (0-0)	0.7 (0-1.5)	0.0 (0-0.7)
**Bristol**	6 (6-6.00)	6 (6-6.00)	5 (5-5.00)	5 (5-5.75)	6.00 (5.00-6.00)	6.00 (5.00-6.00)
**pH**	6.065 (5.70-6.90)	7.240 (6.98-7.61)	7.220 (7.07-7.42)	6.600 (6.36-6.93)	6.95 (5.91-7.27)	6.97 (6.57-7.29)
**Total bacteria (log 10) genomes/g**	25.19 (23.98, 29.93)	28.02 (26.43, 29.93)	27.82 (24.75, 28.89)	25.24 (24.47, 26.80)	26.50 (24.40-29.41)	26.63 (25.45-28.40)

Tested biomarkers: Intestinal alkaline phosphatase (IAP), bactericidal/permeability-increasing protein (BPI), human alpha defensin 5 (HD5), eosinophil cationic protein (ECP), immunoglobulin A (IgA), lactoferrin (LTF), albumin (Alb), lipocalin 2 (LCN2), calprotectin (Cal), mucin 2 (Muc2), Lysozyme and zonulin, total protein and total bacterial levels, pH, and Bristol score. All data points were included.

### DNA extraction and qPCR

Bacterial DNA was extracted from faecal samples using a modified version of repeated bead beating^[25]^. Briefly, the faecal DNA was extracted from 250 to 340 mg of faecal material that was suspended in 0.5 mL of sterile ice-cold phosphate-buffered saline (PBS), and 250 μL of the faecal suspension was combined with 340 μL of RBB lysis buffer [500 mM NaCl, 50 mM Tris-HCl (pH 8.0), 50 mM EDTA, 4% SDS] in a bead-beating tube from the Ambion MagMAX^TM^ Total Nucleic Acid Isolation Kit (Life Technologies). After three rounds of repeated bead-beating in a FastPrep®-96 instrument (MP Biomedicals, Santa Ana, CA, United States) at a speed of 800 rpm for 60 s, the lysate was collected. A second round of bead beating was conducted with 145 μL of fresh RBB buffer, repeating 3 times for 60 s each, to lyse the remaining intact cells. Pooled supernatant (250 μL) was used for DNA extraction with a KingFisher^TM^ Flex automated purification system (ThermoFisher Scientific) using a MagMAX^TM^ Pathogen High Vol. DNA was quantified using Quanti-iT^TM^ Pico Green dsDNA Assay (Invitrogen)^[26]^.

Quantification of total bacteria was carried out by qPCR using a BioRad iCycler iQ thermal cycler system (BioRad, Hercules, CA) with HOT FIREPol® EvaGreen® qPCR Mix Plus (Solis BioDyne, Tartu, Estonia) as explained^[12]^, 331F (TCCTACGGGAGGCAGCAGT)/797R (GGACTACCAGGGTATCTAATCCTGTT) primers targeting the 16S rRNA gene and 0.5 ng of faecal DNA. Briefly, the thermal cycling conditions started with a DNA-denaturation step at 95 °C for 15 min, followed by 40 cycles of (1) denaturation at 95 °C for 15 s; (2) annealing at a primer-specific temperature for 20 s; (3) extension at 72 °C for 30 s; and (4) an incubation step to detect the fluorescent data. A melting curve analysis was carried out to ensure the specificity of the amplification products. The 10-log-fold standard curves ranging from 10^2^ to 10^7^ copies were produced using the full-length amplicons of the 16S rRNA gene of *Bifidobacterium longum* to convert the threshold cycle (Ct) values into the average estimates of genomes present in 1 g of faeces (copy numbers/g of wet faeces) in each assay^[26]^.

### Metagenomic sequencing

Sequencing libraries were prepared using the Illumina Nextera DNA Flex kit, according to the manufacturer’s instructions. Shotgun metagenomic sequencing of 2 × 150 bp was performed with an Illumina NovaSeq system using S4 flow cells with a lane divider (Illumina, San Diego, CA, United States) at the sequencing laboratory of the Institute for Molecular Medicine Finland (FIMM), University of Helsinki.

### Taxonomic annotation

Raw reads were filtered using fastp^[27]^, with parameters Q < 20, min read length > 50, reads merged with a minimum of 15 bp overlap, reads with Ns discarded, and 3 bp trimmed from the front and back of the reads. To remove host DNA, filtered reads were mapped using Minimap2 (Li, 2021) and SAMtools^[28]^ against the human genome (GRCh38.p14, NCBI RefSeq assembly: GCF_000001405.40). Taxonomic annotation was performed by mapping the filtered reads using Mininimap2 against the HumGutDB^[29]^. Relative abundances were summarized at different taxonomic levels in R and translated into absolute abundances by multiplying with the total number of bacterial genomes.

### Bristol score

Bristol score was determined visually from faecal samples upon retrieval from -20 °C storage.

### Faecal water extraction from faecal samples

Approximately 100 mg aliquots of the frozen faecal samples were taken and suspended into 0.5 mM solution of PMSF protease inhibitor (Thermo Scientific, 36978) in 1× PBS (VingLab) in a 1:10 ratio. The median pH was 6.97 (interquartile range 6.47-7.28). Samples were kept on ice. Samples were centrifuged twice for 15 min, at +4 °C at 13,000 rpm. Sample supernatants were distributed to 96-well plates for storage at -80 °C and further analysis.

### Biomarker quantification

All absorbances were read with Hidex Sense microplate reader.

#### Total protein levels

Total protein concentrations were measured using a DC Protein Assay Reagents Package (5000116, Bio-Rad) according to the manufacturer’s protocol, with bovine serum albumin lyophilized (Biowest, P6154) as standard. A linear standard curve was used for the calculation of the results.

#### Albumin - Alb

Albumin was quantified with sandwich ELISA using Human Albumin Matched Antibody Pair Kit (Abcam, ab246841) according to the manufacturer’s general protocol for matching antibody pair kits. A four-parameter logistic curve was used for the calculation of the results.

#### IAP

IAP activity was quantified with QUANTI-Blue solution (rep-qbs, InvivoGen) according to the manufacturer’s instructions. A standard test was performed to identify the correct standard concentrations. Secreted embryonic alkaline phosphatase (SEAP) protein (rec-hseap, Invivogen) was used as the standard. A linear standard curve was used for the calculation of the results.

#### BPI

BPI was quantified by sandwich ELISA using BPI, Human, ELISA kit (HK314, Hycult Biotech) according to the manufacturer’s instructions, except samples were diluted 1:2 in dilution buffer. A four-parameter logistic curve was used for the calculation of the results.

#### HD5

Alpha defensin five was quantified with sandwich ELISA using HD5 (Paneth Cell Specific) ELISA Kit (E-EL-H1798, Elabscience) according to the manufacturer’s instructions. Samples were diluted 1:2 in reference standard and sample diluent. A four-parameter logistic curve was used for the calculation of the results.

#### ECP

ECP was quantified with sandwich ELISA using Human RNASE3/ECP (Ribonuclease A3/Eosinophil Cationic Protein) ELISA Kit (E-EL-H1379, Elabscience) according to the manufacturer’s instructions. Samples were diluted 1:2 in reference standard and sample diluent. A four-parameter logistic curve was used for the calculation of the results.

#### Muc2

Muc2 was quantified by sandwich ELISA using Human Muc2 ELISA Kit (E-EL-H0632, Elabscience) according to the manufacturer’s protocol. Samples were diluted 1:4 in reference standard and sample diluent. A four-parameter logistic curve was used for the calculation of the results.

#### IgA

IgA was quantified by sandwich ELISA using Human IgA Matched Antibody Pair Kit (Abcam, ab219536) according to the manufacturer’s general protocol for matched antibody pair kits. A linear standard curve was used for the calculation of the results.

#### LTF

LTF was quantified with sandwich ELISA using Human Lactotransferrin / LTF ELISA Pair Set (SEK11096, SinoBiological) according to the manufacturer’s instructions. A linear standard curve was used for the calculation of the results.

#### LCN2

LCN2 was quantified with sandwich ELISA using Human Lipocalin-2/NGAL Matched ELISA Antibody Pair Set (SEK10222, SinoBiological) according to the manufacturer’s instructions, using 100 μL of STOP solution per sample. A linear standard curve was used for the calculation of the results.

#### Cal

Cal was quantified by sandwich ELISA using Human Cal (S100A8 + S100A9) Antibody Pair - BSA and Azide free (Abcam, ab309558) and Recombinant Human Cal (S100A8 + S100A9) protein (Abcam, ab130945) for the standards. The detector antibody was biotinylated using Biotinylation Kit / Biotin Conjugation Kit (Fast, Type A) - Lightning-Link® (Abcam, ab201795) according to the manufacturer’s instructions. The ELISA was performed according to the general matched antibody pair kit protocol in Human IgA Matched Antibody Pair Kit (Abcam, ab219536). A linear standard curve was used for the calculation of the results.

#### Lysozyme

Lysozyme was quantified by sandwich ELISA using Human Human LZM (Lysozyme) ELISA Kit (Elabscience, E-EL-H1869). A four-parameter logistic curve was used for the calculation of the results.

#### Zonulin

Zonulin was quantified by sandwich ELISA using Human Zonulin ELISA Kit (elabscience, E-EL-H5560) according to the manufacturer’s instructions. A four-parameter logistic curve was used for the calculation of the results.

### Statistical analysis

All analyses were performed in R 4.3.1 (2023-06-16) within Rstudio Version 2023.06.2+561 for macOS.

We used R the packages mare^[30]^, reshape2^[31]^, nlme^[32]^, and gplots^[33]^. Both microbial absolute abundances and biomarker levels were analysed after log transformation to obtain normal distributions. We calculated the daily changes in both by subtracting the log-transformed abundance on day t from the log-transformed abundance on day t + 1. Bacteria were analysed at the family and genus levels, including only taxa that were present at > 0.1% in at least 50% of at least one of the time series (48 genera and 23 families). We used linear mixed models (function lme) to identify associations between microbial taxa and biomarkers, with the time series ID, combining information on subject ID and age, as a random factor. The model residuals were random and normally distributed without temporal patterns. The models were adjusted for total protein concentration/change and total bacteria change in the sample.

When modelling the associations between biomarker concentrations and bacterial changes, the biomarker concentrations were normalised by scaling and centring by time series to remove average-level differences between individuals. Due to the exploratory nature of the study, we chose to define statistical significance as *P* < 0.05 without multiple testing adjustment, as it was considered important to find all true associations (reduce the number of false negatives) even if some false positives may arise. To reduce the number of false positives, we did not analyse the data at the species level, as we expect related species to have similar biomarker associations.

## RESULTS

We investigated associations between faecal microbiota and immune-related biomarkers in daily time series of 6 infants sampled at the age of 5-6 and/or 11-12 months. The infants were selected from a larger cohort for this exploratory study based on diverse microbiota compositions, including *Bifidobacterium*-dominated, *Bacteroides*-dominated, *Enterobacteriaceae*-dominated, and Clostridia-dominated communities to maximize the generality of the results despite the small sample size. All infants were breastfed at 5-6 months and ¾ infants at 11-12 months.

### Biomarker concentrations

The average immune-related biomarker concentrations fluctuated during each time series, but typically did not show strong directional change. They did not differ by birth mode or infant age, apart from Cal, which was higher in the caesarean-born infants (*P* = 0.02, linear mixed model, Table 1), and Bristol score, which was higher at 6 months (*P* < 0.001).

### Associations between faecal biomarkers

**Figure 1:**
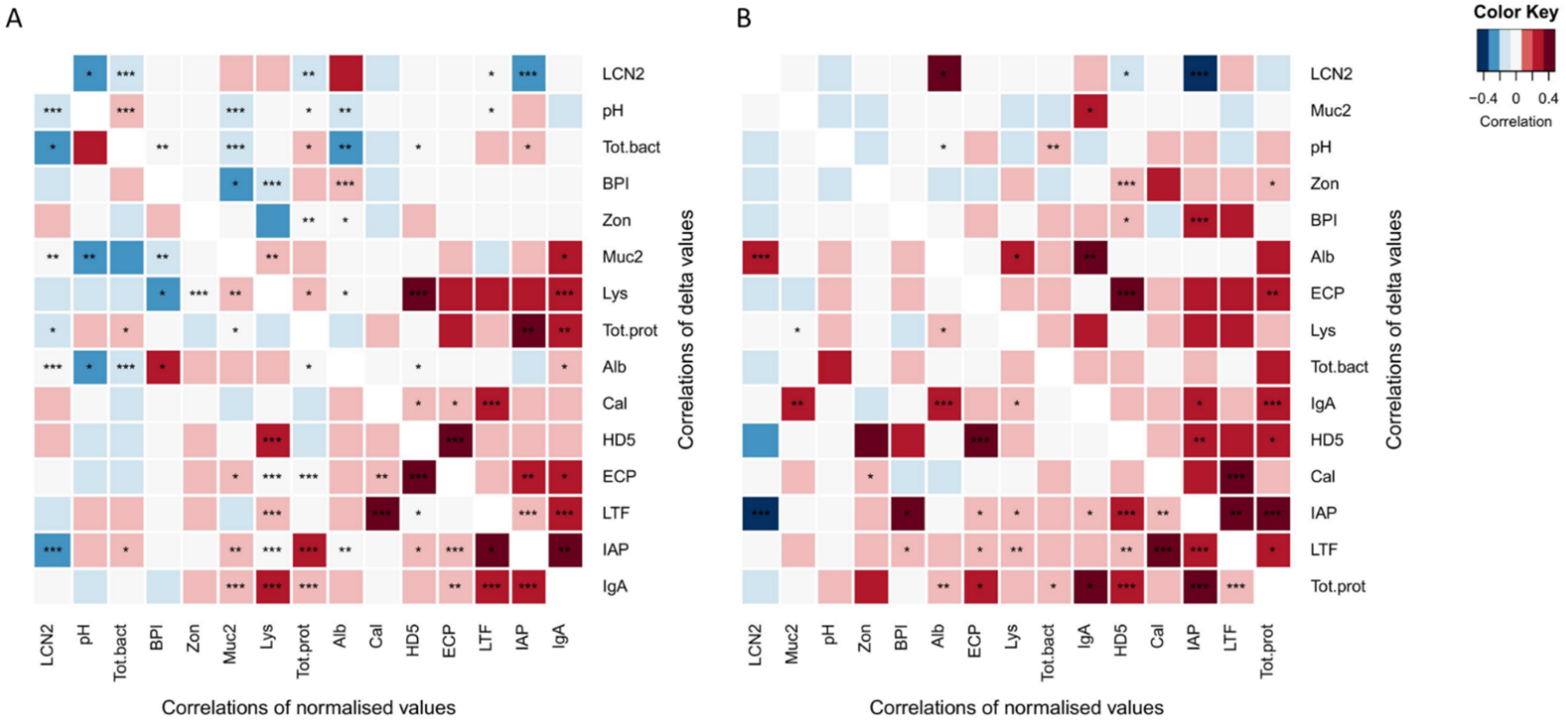
Intercorrelations between faecal biomarker concentrations, lower left triangle, and daily changes (delta), upper right triangle, (A) at 6 months and (B) at 12 months. *P*-values are indicated as asterisks: *P* < 0.001^***^; *P* < 0.1^**^; *P* < 0.5^*^.

As a first exploratory step, we assessed the raw correlations between faecal biomarkers normalised within each time series without adjusting for repeated sampling. At both 6 and 12 months, total bacteria load was positively associated with pH and total protein [Figure 1A and B]. The concentrations of most measured biomarkers - IAP, LTF, Cal, Muc2, HD5, lysozyme, and ECP - were positively correlated with each other and with total protein and total bacterial load [Figure 1A]. BPI was positively correlated with albumin and negatively correlated with lysozyme and Muc2 [Figure 1A]. At 12 months, total protein was still significantly correlated with IAP, LTF, HD5, IgA, and ECP and with Cal, but not with Cal [Figure 1B]. Cal correlated only with zonulin and vice versa [Figure 1B]. Total bacteria were not significantly associated with any biomarker at 12 months [Figure 1B].

At both time points, LCN2 concentrations were inversely correlated with the protein and bacteria content of the stool and with pH, but only significantly at 6 months [Figure 1A and B]. Muc2 was negatively associated with pH at 6 months and with total bacteria at both time points [Figure 1A and B].

To obtain a more detailed understanding of the interrelated dynamics between the biomarkers, we analysed the associations between their daily changes [Figure 1A and B upper right triangle]. Increases in total bacterial load were associated with increasing pH and total protein content at both time points. Changes in HD5 were positively correlated with changes in ECP, BPI, and zonulin. So too were changes in albumin and IgA, and LTF, IAP, and Cal [Figure 1A and B].

Changes in LCN2 were negatively correlated with changes in IAP, total protein, and pH at 6 months. At 6 months, muc2 decreased when total bacteria or pH increased [Figure 1A]. These associations were still present but not significant at 12 months [Figure 1B].

Because bifidobacteria have been previously associated negatively with faecal pH in infants, we tested these associations specifically, and discovered a significant negative association at 6 months (*P* ≤ 0.001), but a positive one at 12 months (*P* ≤ 0.001).

### Associations between bacterial population growth and faecal biomarker changes

#### General association patterns

**Figure 2:**
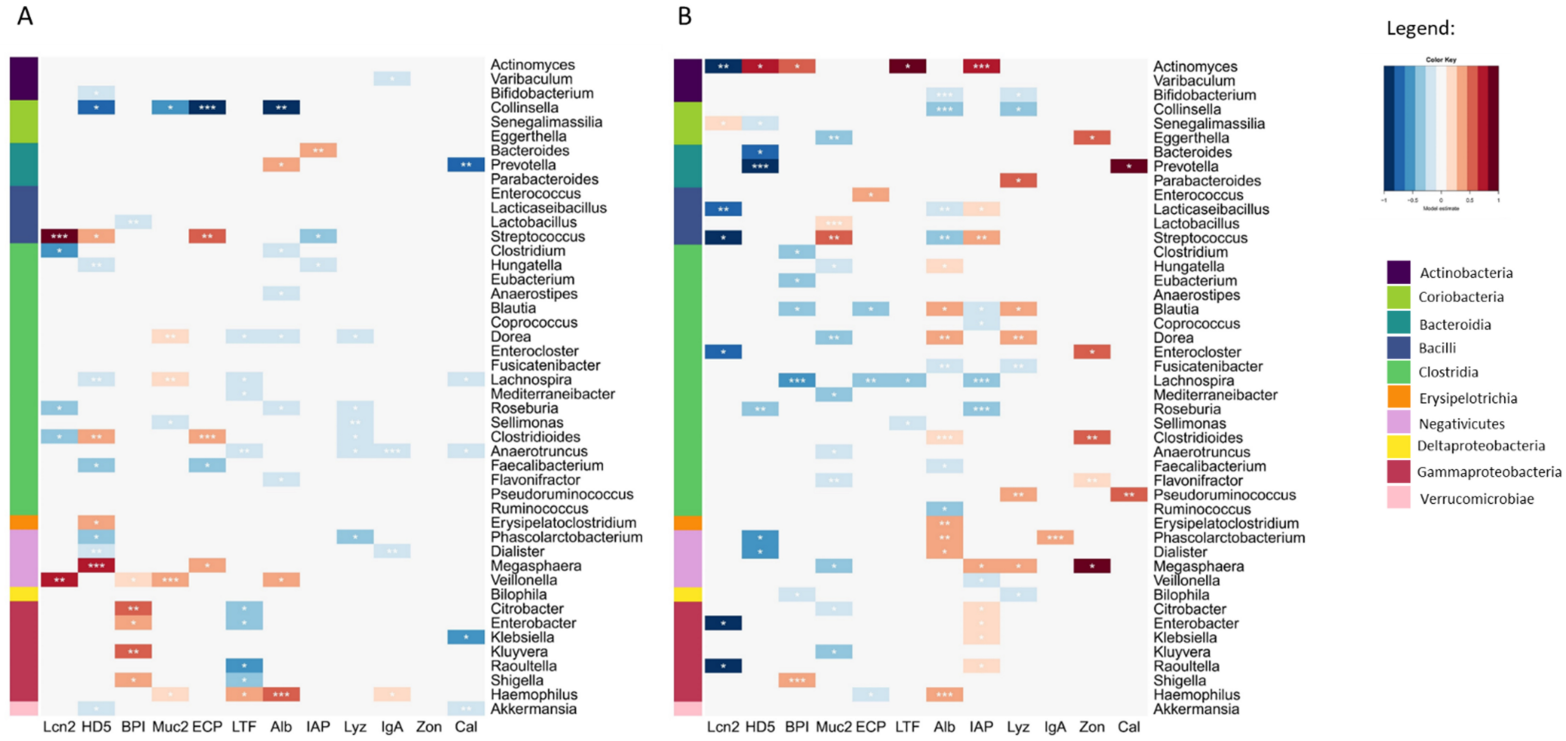
Gut microbes predicting faecal biomarker changes after adjustment for total protein and total bacteria change. Associations between daily faecal biomarker changes and daily changes in gut microbe abundances at genus levels at (A) 6 months and (B) 12 months of age. The colour represents the strength of association from a linear mixed model adjusting for total protein and total bacterial abundance changes. *P*-values are indicated as asterisks: *P* < 0.001^***^; *P* < 0.1^**^; *P* < 0.5^*^. The microbial class is presented as the sidebar colour.

We attempted to identify bacterial stimulation of immune biomarkers by predicting the daily change in biomarker concentrations with the daily changes in microbial absolute abundances. We identified both negative and positive associations between bacterial population growth and biomarker changes, potentially indicative of bacterial stimulation or inhibition of biomarker expression [Figure 2]. Overall, *Collinsella* emerged as the most consistent and the strongest potential inhibitor of many immune-related biomarkers, especially ECP. *Bifidobacterium* and *Akkermansia* stood out due to their weak and exclusively negative associations with the biomarkers [Figure 2]. Both had a negative association with HD5 and Cal at 6 months (although not significant for *Bifidobacterium*), and *Bifidobacterium* with albumin at 12 months.

#### Indicators of gut homeostatic regulation

Levels of muc2 at 6 months increased with an increase in *Haemophilus*, *Veillonella*, *Lachnospira*, and *Dorea*, and decreased with increasing levels of *Sellimonas* and *Collinsella* [Figure 2A]. At 12 months, muc2 decreased with an increase in many bacterial genera, including members of Clostridia, but was positively associated with *Lactobacillus* and *Streptococcus* [Figure 2B]. Albumin levels declined with increasing *Collinsella* (both time points) and *Bifidobacterium* (12 months) and increased in association to *Haemophilus* and members of Negativicutes [Figure 2A and B]. IgA had mostly negative associations at 6 months, including with *Anaerotruncus* and *Dialister*, but mostly positive ones at 12 months, with *Phascolarctobacterium* statistically significant [Figure 2A and B].

IAP was positively correlated with *Bacteroides* at 6 months [Figure 2A], and *Actinomyces*, *Streptococcus*, and genera of the *Enterobacteriaceae* family at 12 months [Figure 2B]. At 12 months, IAP had negative associations with members of Clostridia, including *Roseburia* and *Lachnospira* [Figure 2B]. BPI was positively associated with members of *Enterobacteriaceae* at both time points and negatively with *Lactobacillus* at 6 months and members of Clostridia at 12 months [Figure 2].

#### Indicators of inflammation

Cal had only negative (*Prevotella* and *Akkermansia*) associations with bacteria at 6 months [Figure 2A], but mostly positive ones at 12 months, mainly with *Prevotella* and *Pseudoruminococcus* [Figure 2B]. LTF had negative associations with members of Clostridia at both time points [Figure 2A and B], and with members of *Enterobacteriaceae* at 6 months [Figure 2A].

Apart from a strong negative association between ECP and *Collinsella*, ECP appeared mostly stimulated by bacteria at 6 months, especially by *Streptococcus* and *Clostridioides* [Figure 2A], while it had mostly negative associations with increasing bacterial populations at 12 months, including *Blautia*, *Lachnospira*, and *Haemophilus* [Figure 2B].

Strong associations were observed for LCN2. At 6 months, increasing abundances of *Streptococcaceae* and *Veillonellaceae* were associated with increasing LCN2 levels, while members of the class Clostridia and *Roseburia* correlated negatively with LCN2 [Figure 2A]. However, these associations were not observable at 12 months [Figure 2B]. At 12 months, LCN2 increase was associated with declining populations of Bacilli and *Enterobacteriaceae* [Figure 2B]*.*

Levels of HD5 were negatively associated with several bacterial taxa, including *Dialister* at both time points, *Bifidobacteriaceae* and *Ruminococcaceae* at 6 months [Figure 2A], and *Prevotellaceae* at 12 months [Figure 2B]. HD5 was positively associated with *Megasphaera* at 6 months [Figure 2A], and with *Actinomyces* at 12 months [Figure 2B].

Lysozyme levels were strongly negatively associated with Clostrida members at 6 months [Figure 2A]. Yet, at 12 months, lysozyme levels were mostly positively correlated with other Clostridia members, *Megasphaera*, *Pseudoruminococcus*, *Parabacteroides*, and *Dorea* but negatively with *Collinsella* and *Bifidobacterium* [Figure 2B].

Zonulin was not associated with any bacteria genera at 6 months, but at 12 months, was positively correlated with several, including *Megasphaera*, *Eggerthella*, and several Clostridia members [Figure 2A and B].

### Associations between biomarker concentration and bacterial population growth

**Figure 3:**
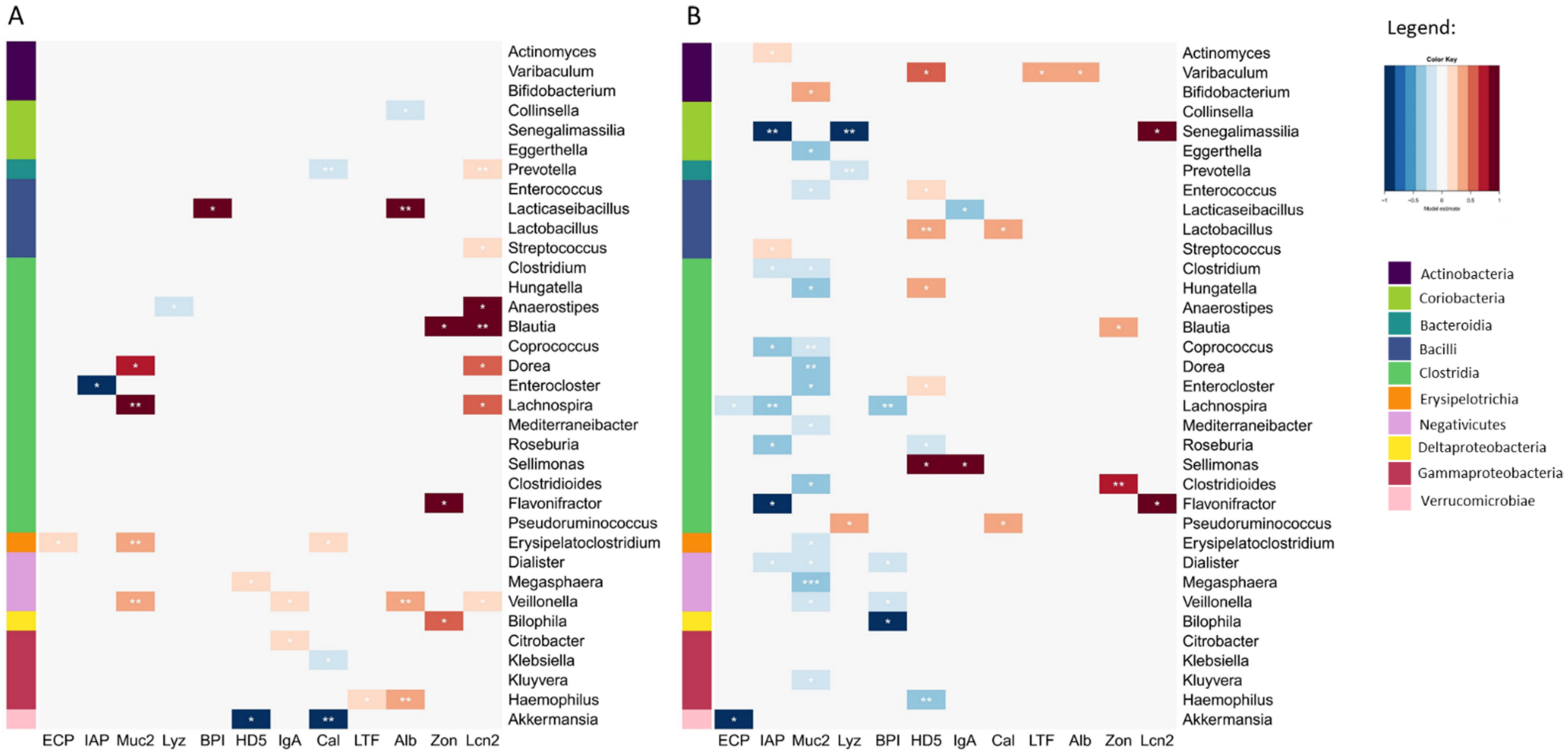
Biomarker levels predicting gut microbe changes. Associations between faecal biomarker concentrations and daily changes in gut microbe abundances at the family and genus levels at (A) 6 months and (B) 12 months of age. The colour represents the strength of association from a linear mixed model adjusting for total protein concentration. *P*-values are indicated as asterisks: *P* < 0.001^***^; *P* < 0.1^**^; *P* < 0.5^*^. The microbial class is presented as the sidebar colour.

We hypothesized that associations between biomarker concentration on day t and the change in bacterial population size from day t to t + 1 (population growth) would represent bacterial responses to the biomarkers [Figure 3].

#### Indicators of gut homeostasis

The association between muc2 and bacterial growth changed from mostly positive at 6 months [Figure 3A] to mostly negative at 12 months [Figure 3B]. The only exception at 12 months was *Bifidobacterium*, which appeared to be stimulated by muc2 [Figure 3B]. At 6 months, higher albumin levels were predictive of increasing *Haemophilus* and *Lacticaseibacillus* [Figure 3A], but no associations were observed at 12 months. IgA at 6 months was predictive of increasing *Veillonella* and *Citrobacter* [Figure 3A], while at 12 months, *Lacticaseibacillus* declined in association with high IgA levels [Figure 3B].

High IAP levels were generally associated with bacterial declines, especially among Clostridia. BPI was negatively associated with the growth of *Bilophila*, *Lachnospira*, and members of Negativicutes at 12 months [Figure 3B], but not at 6 months.

#### Indicators of inflammation

Cal was mostly negatively associated at 6 months, notably with *Akkermansia*, *Klebsiella*, and *Prevotella*, and was only positively correlated with *Erysipelatoclostridium* [Figure 3A]. Cal was positively correlated with *Lactobacillus* and *Pseudoruminococcus* at 12 months [Figure 3B]. LTF and ECP concentrations had no strong associations with microbial changes, apart from a notable strong negative association between ECP and *Akkermansia* at 12 months. High levels of LCN2 were associated with increasing abundances of many Clostridia genera at 6 months [Figure 3A], but these were mostly non-significant at 12 months. High levels of HD5 were negatively associated with *Akkermansia* (significant only at 6 months) and *Haemophilus* (12 months), and positively with *Lactobacillus*, *Enterococcus*, and *Enterocloster* (12 months) [Figure 3A and B]. High lysozyme levels predicted a decline in *Anaerostipes* at 6 months. At 12 months, it correlated with increasing levels of *Pseudoruminococcus* and decreasing levels of *Prevotella* and *Senegalimassilia.* A high zonulin level was associated with increasing abundance of *Blautia* at 6 and 12 months[Figure 3A and B]*.* At 6 months, it was also associated positively with *Bilophila and Flavonifractor* [Figure 3A], and at 12 months with *Clostridioides* [Figure 3B].

## DISCUSSION

Utilising densely sampled absolute abundance time series of infant gut microbiota and immune-related biomarkers, we were able to identify potential host-microbe interactions occurring *in vivo* in healthy infants. Although microbial programming of the immune system is considered important during early life, there is limited research on this topic in human infants. Most of the previous work on the subject has been done *in vitro* or in mice. Exploring approaches to investigate host-microbe interactions in humans *in vivo* is important, as *in vitro* studies often do not represent the whole human gut ecology^[34]^ and mouse models suffer from questionable relevance and difficulties in interpretation and translation^[35,36]^.

At 6 months, most immune biomarkers were intercorrelated and correlated to total protein, total bacterial content and pH, except BPI, albumin, and LCN2, which were associated with each other and inversely correlated with bacterial load and pH. The intercorrelated markers included LTF, IgA, and lysozyme, which, especially at 6 months, may be derived from breastmilk^[37]^. However, due to the strong associations with the non-breastmilk-associated biomarkers, such as ECP, DH5, and Cal^[35]^, it is likely that the LTF and IgA in our samples are mostly infant-derived.

In infants, low pH has been associated with a high abundance of bifidobacteria^[38,39]^, which we confirmed at 6 months when bifidobacteria are typically the dominant group and the most important taxon responsible for human milk oligosaccharide fermentation, but not at 12 months when the fermentation of other, non-HMO substrates will be more prevalent.

We identified indications of microbe-induced stimulation of muc2, ECP, IgA, albumin, Cal, HD5, IAP, and BPI at both time points, as well as stimulation of LCN2 and LTF only at 6 months. The associations between biomarker concentrations and bacterial population growth were mostly positive at 6 months and largely negative at 12 months, suggestive of increasing host regulation of the microbiota with age. The results indicate an effect of immune signalling in the gut shifting from tolerance toward more defensive, as the immune system does when maturing^[7,40]^. The exception was Cal, whose effects changed from negative at 6 months to positive at 12 months. Thus, the major host-derived regulators of microbial growth appeared to be Cal at 6 months, and IAP, BPI, and mucin at 12 months. We will briefly discuss the results per biomarker below.

### Markers of gut homeostatic regulation

Muc2 is the most abundant mucin in the gut and, thus, an important mediator of host-microbe interactions^[41]^. We hypothesised that faecal muc2 may indicate the balance between mucus production and degradation and, thus, the condition of the gut mucus layer. Our results suggest that mucin degradation is more substantial at 6 months than at 12 months, as muc2 decreased with increasing bacterial load only at 6 months, and it appeared to stimulate microbial growth at 6 months but to reduce it at 12 months. Before infants consume substantial amounts of solid foods, breastmilk and mucin are the primary carbon sources for gut bacteria^[41]^. *In vivo*, the mucin, which forms the intestinal epithelial mucous membrane, would serve as a scaffold and source of nourishment for bacteria when the infant is not yet weaned^[41]^. Negative associations between muc2 and members of Clostridia were observed at both time points, suggesting that these organisms either reduce its secretion or participate in its degradation. The former is more likely since these populations did not appear to benefit from increasing muc2 levels.

At 12 months, muc2 emerged as a potential inhibitor of microbial growth. Only *Bifidobacterium* appeared to benefit from muc2 at 12 months. Mucin contains a similar oligosaccharide structure as breastmilk, and therefore, some of the breastmilk-adapted *Bifidobacterium spp*. can also degrade mucin^[42]^. Thus, *Bifidobacterium spp*. likely benefit from increasing muc2 levels, especially at 12 months, when the amount of breastmilk the infant receives is decreasing. We did not observe a significant association between muc2 and the mucin-degrading *Akkermansia*, possibly because *Akkermansia* grows in the mucus layer and its abundance may not depend on luminal mucin.

Albumin is the most abundant protein in blood, and thus, its presence in faecal samples has been suggested to be an indication of increased gut permeability^[23]^. Albumin increased in association with Gram-negative organisms, including *Haemophilus*, but lowered with *Collinsella* and *Bifidobacterium.* These results are in line with previous findings from preterm infants, where increased gut permeability measured by the lactulose-mannitol test was associated with opportunistic pathogens, such as *Staphylococcus* and enterobacteria, while permeability was low in infants dominated by bifidobacteria^[43]^. Albumin was correlated with IAP and BPI, suggesting a shared regulatory pattern or the possibility that increased permeability stimulates the secretion of antibacterial compounds. However, zonulin, a protein known to increase tight junction permeability and often used as a marker of gut permeability^[44]^, did not correlate with albumin. In most infants, zonulin levels were below the detection level. Our results suggest that albumin may be a more sensitive marker of gut permeability in healthy infants than zonulin.

Secretory IgA is the most abundant antibody in the intestine and is considered the first line of defence against pathogens in the gut^[21,45,46]^, but is also secreted into breastmilk. IgA is thought to help support favourable microbiota^[21,46]^. We found that changes in IgA correlated with changes in IAP, lysozyme, and albumin. Like albumin, IgA was positively associated with *Haemophilus* at 6 months and at 12 months by *Phascolarctobacterium*. Putative inhibition or degradation of IgA by specific bacterial populations was observed at 6 months but not at 12 months.

IAP is produced by intestinal epithelial cells in response to bacterial lipopolysaccharide (LPS) produced by Gram-negative bacteria, and it serves to neutralise LPS and thus limit LPS-induced inflammation^[15,47,48]^. Alkaline phosphatase is also produced by bacteria, and it has been estimated that 20%-30% of faecal alkaline phosphatase activity is derived from bacteria^[49,50]^. Our IAP measurement may have suffered from neutral pH, as its activity is optimal at pH 10, but dilution with PBS rather than an alkaline buffer enabled the simultaneous analysis of multiple biomarkers from the same faecal water sample. In our data, IAP levels correlated with other markers of inflammation, but IAP increased together with IgA and LTF - molecules whose role is to limit inflammation by binding and eliminating microbes and antigens. A correlation between IAP and IgA has been shown before in mice and humans^[50]^, suggesting that they may be regulated by common factors, or as suggested by Lassenius *et al*., immunoglobulin secretion may be stimulated by IAP^[50]^. *Bacteroides* appeared as the main putatively stimulatory bacterium of IAP at 6 months, and members of Proteobacteria and Negativicutes at 12 months. IAP counters the inhibitory effect of ATP on bacterial growth and is believed to affect the balance of gut microbes, as IAP KO mice are not colonised with *Escherichia coli* and have increased Clostridia levels^[47,51,52]^*.* Our data confirm similar associations in human infants. IAP thus emerges as a potential regulator of gut microbiota in human infants, appearing to be stimulated by Gram-negative bacteria and to inhibit the growth of Gram-positive bacteria.

BPI is a microbicidal protein with endotoxin-neutralising abilities produced by neutrophils^[16]^. We found BPI to be generally stimulated by the overall bacterial load, specifically by Proteobacteria, while its expression appeared to be attenuated by *Lactobacillus* at 6 months and by members of Clostridia at 12 months. BPI appeared to inhibit bacterial growth only at 12 months, when it was especially effective against *Bilophila*, known for its inflammatory effects^[53]^. BPI’s generally negative associations with bacterial growth fit its function as a microbicidal peptide. Our data indicate that BPI activity may mature after the introduction of solid foods, as it is not stimulated by high bacterial abundance nor inhibitory against bacteria at 6 months.

### Markers of inflammation

Cal is a calcium-, zinc-, and manganese-binding protein secreted by neutrophils. It competes with bacteria (and fungi) for metals, thereby inhibiting their growth^[19]^. So far, it has been shown to inhibit the growth of diverse pathogens, including *S. aureus*, *Acinetobacter baumannii*, *Helicobacter pylori*, *Salmonella enterica*, *Staphylococcus epidermidis*, *Staphylococcus lugdunensis*, *Enterococcus faecalis*, *Pseudomonas aeruginosa*, and *Shigella flexneri*^[54]^*.* It is also proposed to inhibit bacterial binding of *L. monocytogenes* and *S. enterica*^[55]^*.* Its abundance in faeces is a marker of inflammation and IBD^[56,57]^. Most literature agrees that Cal levels in healthy infants are higher than in adults due to many factors, including fluctuation of microbiota^[58]^. In fact, lower Cal levels are associated with dysregulated microbiota and feeding intolerance in infants^[57]^. The levels tend to drop with age, stabilising at adult levels around a year of age^[59]^. We found higher Cal levels in C-section-born infants compared to vaginally born infants [Table 1]. Different studies have found higher Cal levels either in C-section-born infants^[60]^ or vaginally born babies^[58,61]^. The differences may be due to differences in gut microbiota compositions, since we found faecal Cal to respond to microbial populations.

At 6 months, *Lachnospiraceae* and *Akkermansia* appeared to inhibit the secretion of Cal, while *Akkermansia* growth seemed to be inhibited by high Cal levels. Bifidobacteria were also negatively associated with Cal levels (albeit not significantly, therefore not shown), which is clinically relevant as supplementation with bifidobacteria has been proposed as a Cal-lowering intervention in babies^[62]^. At 12 months, *Pseudoruminococcus* and *Prevotella* appeared to stimulate Cal, and Cal seemed to have only positive effects on bacterial growth - most strongly on *Pseudoruminococcus and Lactobacillus.*

LTF is an iron-sequestering protein with antibacterial and antiviral activity that is found in breastmilk and is secreted by neutrophils in the gut in response to inflammation^[20,63]^. Because we found LTF to correlate with Cal, most of the LTF in our samples is likely gut-derived rather than originating in breastmilk. LTF has been proposed to reduce inflammation, neutralise endotoxins, and, most importantly, aid in commensal colonisation^[63-65]^. However, most of these studies are done *in vitro* or look at immune biomarkers rather than bacteria. Apart from *Haemophilus* at 6 months and *Varibaculum* at 12 months, we did not observe significant effects of LTF on gut microbes. This has also been reported after administering oral LTF to toddlers^[66]^.

HD5 is an antimicrobial peptide produced by Paneth cells, known to selectively kill pathogens and preserve commensals^[17]^. It is increased in the inflamed colon of children with IBD and associated with atopic dermatitis^[67,68]^. We found that it correlates with ECP, suggestive of a common regulatory system, although ECP is produced by eosinophils^[18]^. Bacteria with known anti-inflammatory properties, such as *Bifidobacterium* (6 months) and *Roseburia* and *Prevotella* (12 months), appeared to reduce its expression, while *Streptococcus*, *Megasphaera*, and *Clostridioides* (6 months) appeared to stimulate it. Lactate has been shown to inhibit HD5^[69]^, but we did not find associations between lactic acid bacteria and HD5. At 6 months, high levels of HD5 appeared to have a negative impact on *Akkermansia*. The association was not significant at 12 months, when high HD5 appeared to reduce the growth of *Haemophilus*, which contains potential pathogens^[70-72]^. In addition, at 12 months, HD5 appeared to stimulate the growth of several commensal bacteria, including *Lactobacillus*.

LCN2 is an iron-sequestering protein secreted by many cell types in the gut, including neutrophils, that inhibits bacterial growth by competing for iron and also has inflammatory effects^[73]^. LCN2 behaved generally differently from the other neutrophil-secreted markers, changing inversely with IAP. Previously, LCN2 has been shown to correlate with Cal in adult IBD patients^[74]^, which we did not observe in this sample of healthy infants. Due to the association of LCN2 with low protein and bacteria concentrations and low pH, as well as with increasing abundances of small intestine organisms (*Veillonellaceae* and *Streptococcacae*^[75]^) and decreasing abundances of colon-dwelling organisms (*Roseburia*^[75]^), we suggest that LCN2 may be a potential indicator of fast gut transit in infants.

ECP is an eosinophil granulocyte-secreted protein that mediates the inflammatory response of the host to microbes and parasites and is elevated in the serum of individuals with atopic diseases such as allergic rhinitis and asthma^[18]^. Levels of ECP also correlate with the disease severity of ulcerative colitis^[76]^. It is cytotoxic by inducing apoptosis yet is also involved in immune modulation and tissue repair^[18]^. ECP has antibacterial effects^[18]^. In our data, it correlated with IAP and HD5. ECP appeared to be stimulated by *Streptococcaceae* and *Clostridioides*, while *Collinsella* seemed to reduce its expression at 6 months. Associations at 12 months were weaker, but *Enterococcus* appeared to stimulate it and some members of Clostridia and *Haemophilus* to inhibit it. ECP seemed to inhibit *Akkermansia* growth at 12 months. Lysozyme is an antimicrobial peptide that cleaves peptidoglycan, the major component of Gram-positive bacterial cell walls^[77]^. It is an important component of human breastmilk, yet it has seemingly conflicting properties: both increased and decreased levels have offered protection against colitis in previous studies^[78-80]^. The effect of lysozyme levels is, therefore, likely to depend on the microbiota. Changes in lysozyme levels were associated with bacterial population growth. At 6 months, bacterial growth appeared to inhibit lysozyme, while at 12 months, we observed both stimulatory and inhibitory associations. Lysozyme has been linked with increased IgA in piglets^[81]^, which we confirmed. While lysozyme has been associated with increasing *lactobacilli* in piglets, we did not find lysozyme to be a strong regulator of the microbiota in human infants^[79,81,82]^.

Zonulin is a protein that reversibly increases the permeability of tight junctions and serves as a biomarker for intestinal barrier integrity^[83]^. Elevated zonulin levels have been associated with many intestinal diseases where barrier dysfunction is involved, such as celiac disease, IBD, and necrotising enterocolitis^[84]^. Its correlation with gut microbiota has been studied in a variety of different settings, and it is elevated in combination with higher levels of gram-negative strains or opportunistic pathogens, such as *Clostridium*, while a higher prevalence of gram-positive bacteria is linked to lower zonulin levels^[84]^. In our data, zonulin appeared to be stimulated by several Gram-positive genera at 12 months, but not at 6 months. On the other hand, zonulin appeared to stimulate the growth of *Flavonifractor*, *Bilophila*, and *Blautia*, suggesting that these bacteria may benefit from increased permeability or inflammation.

### Limitations

This research has analysed 216 samples so far. According to power calculation, with a sample size of 216, we could detect a correlation coefficient of 0.19 or larger at the 0.05 *P*-value cut-off and with a 0.2 type II error rate. The sample size is thus sufficient to detect modest associations. However, these samples are derived from 8 faecal time series of infants from the same area in Finland, meaning that the correlations found here might not be representative of infants in general and would need to be replicated in an independent cohort to test reproducibility and generalisability of the found associations. Most (3 out of 4) 11-12-month-olds were still breastfed, and we, therefore, do not have sufficient representative data for infants who solely consume solid food or formula milk. We did not control diet, and thus, infants present with a natural variation in microbiome based on variations in their diet in addition to any inherent variation between infants. Future research will expand the variety of infants sampled, enabling a more robust correlation of biomarkers, microbiome, and gut health. While we were able to observe consistent associations between microbial population growth and biomarkers’ abundances and changes, these do not enable causal inference.

### Conclusions

Despite the limited sample variety, this study provides a novel perspective on microbiota-host interactions in the infant gut during a critical time of immune system maturation. Our results add a dynamic perspective to host-microbe interactions, suggesting that gut permeability and immune system responses in healthy infants may fluctuate in association with the microbial stimuli, which is likely important in the maintenance of gut homeostasis. We observed a change in the nature of microbe-host interactions from apparent immune tolerance at 6 months toward more tight regulation at 12 months. IAP activity, measured by the Quanti-Blue assay, and muc2, measured by ELISA, emerged as potentially important regulators of gut microbiota in infants. The study demonstrates the utility of biomarker and bacteria profiling from daily stool samples as a potent tool for analysing *in vivo* associations between the immune system and the gut microbiota.

## DECLARATIONS

### Availability of data and materials

The data that support the findings of this study, the taxonomic tables and biomarker concentrations, are available as supplementary data.

### Copyright

© The Author(s) 2024.
